# Radon Exposure and Gestational Diabetes

**DOI:** 10.1001/jamanetworkopen.2024.54319

**Published:** 2025-01-10

**Authors:** Yijia Zhang, Meghan Angley, Liping Lu, Brian J. Smith, William Grobman, Blair J. Wylie, Noelia M. Zork, Mary E. D’Alton, Becky McNeil, Brian M. Mercer, Robert M. Silver, Hyagriv N. Simhan, David M. Haas, George R. Saade, Samuel Parry, Uma Reddy, Ka Kahe

**Affiliations:** 1 Department of Obstetrics and Gynecology, Vagelos College of Physicians and Surgeons, Columbia University Irving Medical Center, New York, New York; 2 Department of Epidemiology, Mailman School of Public Health, Columbia University, New York, New York; 3 Department of Nutrition and Health Science, College of Health, Ball State University, Muncie, Indiana; 4 Department of Biostatistics, College of Public Health, The University of Iowa, Iowa City; 5 Department of Obstetrics and Gynecology, College of Medicine, The Ohio State University, Columbus; 6 Department of Environmental Health Sciences, Mailman School of Public Health, Columbia University, New York, New York; 7 RTI International, Research Triangle Park, North Carolina; 8 Division of Maternal-Fetal Medicine, Department of Obstetrics and Gynecology, Case Western Reserve University School of Medicine, Cleveland, Ohio; 9 Division of Maternal-Fetal Medicine, Department of Obstetrics and Gynecology, University of Utah Health Sciences Center, Salt Lake City; 10 Department of Obstetrics, Gynecology, and Reproductive Sciences, Magee-Womens Research Institute & Foundation, University of Pittsburgh School of Medicine, Pittsburgh, Pennsylvania; 11 Division of Maternal-Fetal Medicine, Department of Obstetrics and Gynecology, Indiana University School of Medicine, Indianapolis; 12 Department of Obstetrics and Gynecology, East Virginia Medical School, Norfolk, Virginia; 13 Division of Maternal-Fetal Medicine, Department of Obstetrics and Gynecology, University of Pennsylvania School of Medicine, Philadelphia

## Abstract

**Importance:**

Understanding environmental risk factors for gestational diabetes (GD) is crucial for developing preventive strategies and improving pregnancy outcomes.

**Objective:**

To examine the association of county-level radon exposure with GD risk in pregnant individuals.

**Design, Setting, and Participants:**

This multicenter, population-based cohort study used data from the Nulliparous Pregnancy Outcomes Study: Monitoring Mothers-to-Be (nuMoM2b) cohort, which recruited nulliparous pregnant participants from 8 US clinical centers between October 2010 and September 2013. Participants who had pregestational diabetes or were missing data on GD or county-level radon measurements were excluded from the current study. Data were analyzed from September 2023 to January 2024.

**Exposures:**

County-level radon data were created by the Lawrence Berkeley National Laboratory based on the Environmental Protection Agency’s short- and long-term indoor home radon assessments. Radon exposure was categorized into 3 groups: less than 1, 1 to less than 2, and 2 or more picocuries (pCi)/L (to convert to becquerels per cubic meter, multiply by 37). Because radon, smoking, and fine particulate matter air pollutants (PM_2.5_) may share similar biological pathways, participants were categorized by joint classifications of radon level (<2 and ≥2 pCi/L) with smoking status (never smokers and ever smokers) and radon level with PM_2.5_ level (above or below the median).

**Main Outcomes and Measures:**

The main outcome was GD, identified based on glucose tolerance testing and information from medical record abstraction. Multiple logistic regression models were used to assess the association between radon exposure and GD.

**Results:**

Among the 9107 participants, mean (SD) age was 27.0 (5.6) years; 3782 of 9101 (41.6%) had ever used tobacco. The mean (SD) county-level radon concentration was 1.6 (0.9) pCi/L, and 382 participants (4.2%) had GD recorded. After adjusting for potential confounders, individuals living in counties with the highest radon level (≥2 pCi/L) had higher odds of developing GD compared with those living in counties with the lowest radon level (<1 pCi/L) (odds ratio [OR], 1.37; 95% CI, 1.02-1.84); after additional adjustment for PM_2.5_, the OR was 1.36 (95% CI, 1.00-1.86). Elevated odds of GD were also observed in ever smokers living in counties with a higher (≥2 pCi/L) radon level (OR, 2.09; 95% CI, 1.41-3.11) and participants living in counties with higher radon and PM_2.5_ levels (OR, 1.93; 95% CI, 1.31-2.83), though no statistically significant interactions were observed.

**Conclusions and Relevance:**

This cohort study suggests that higher radon exposure is associated with greater odds of GD in nulliparous pregnant individuals. Further studies are needed to confirm the results and elucidate the underlying mechanisms, especially with individual-level residential radon exposure assessment.

## Key Points

**Question:**

Is there an association between radon exposure and the risk of gestational diabetes (GD) in pregnant individuals?

**Findings:**

In this national cohort study of 9107 nulliparous pregnant participants, individuals living in US counties with higher radon levels (≥2 picocuries [pCi]/L) had higher odds of developing GD compared with those living in counties with lower radon levels (<1 pCi/L).

**Meaning:**

These findings suggest that higher county-level radon exposure is associated with GD among nulliparous pregnant individuals, highlighting the importance of considering environmental risk factors in maternal health strategies.

## Teaser

This study investigates whether US county-level radon measurements are associated with gestational diabetes among nulliparous pregnant individuals.

## Introduction

Gestational diabetes (GD) is characterized by the development of hyperglycemia during pregnancy.^1^ According to the Centers for Disease Control and Prevention, GD affects approximately 10% of pregnancies annually in the US.^2^ GD has long-term impacts on both women and their offspring, increasing the risk of type 2 diabetes and cardiovascular disease among mothers and childhood obesity.^3^ Apart from known biological risk factors, environmental influences, such as fine particulate matter air pollutants (PM_2.5_) and smoking, have been increasingly recognized in association with GD risk.^4,5^

Radon is a gas formed through the radioactive decay of radium-226, found in soil, rocks, and water.^6,7^ Radon gas decays into solid radon decay products (RDPs) and can enter homes and other buildings through cracks in their substructure.^6,7^ These RDPs are the primary source of naturally occurring radiation exposure in the general population.^6,8,9^ Notably, RDPs can attach to ambient PM, forming radioactive particles that can be inhaled and circulated to various organs and tissues.^10,11^ While it is well established that exposure to radon is associated with an increased risk of lung cancer,^12,13^ other potential health risks are uncertain.

Exposure to PM_2.5_ is associated with an increased risk of GD,^14^ suggesting a potential relationship between radon and GD. One study showed that ambient particle radioactivity was associated with higher risk of GD even after controlling for PM_2.5_.^15^ The association between these radioactive particles and GD may stem from inflammation,^16^ systemic oxidative stress,^17^ and/or insulin resistance^18^—the same mechanisms of the association of maternal smoking with GD.^19^

Epidemiologic evidence directly linking radon exposure to GD is lacking. An ecological study serves as an important initial step, helping to generate hypotheses for future research. Our primary objective was to examine the association between county-level radon exposure and the risk of GD in a large, racially and ethnically diverse sample of pregnant individuals in the US. We also explored the interactions of radon exposure with smoking and PM_2.5_ given the potential shared pathways related to the development of GD.

## Methods

### Study Population and Design

The Nulliparous Pregnancy Outcomes Study: Monitoring Mothers-to-Be (nuMoM2b) is a multicenter, prospective cohort study designed to examine the factors associated with pregnancy-related outcomes. Nulliparous individuals with singleton pregnancies from 8 clinical centers in the US were enrolled in the study between October 2010 and September 2013.^20^ The detailed study design and protocol have been published elsewhere.^20^ In brief, eligible participants were between 6 0/7 and 13 6/7 weeks’ gestation and had no self-reported previous pregnancies lasting 20 weeks or longer. The nuMom2b study was approved by the institutional review boards (IRBs) of all participating institutions, and all included individuals provided written informed consent. The Columbia University IRB approved the current study and waived additional informed consent because data were deidentified. The reporting of the current study followed the Strengthening the Reporting of Observational Studies in Epidemiology (STROBE) reporting guideline for cohort studies.^21^

Study visits (not part of clinical care) occurred in each trimester: 6 0/7 to 13 6/7, 16 0/7 to 21 6/7, and 22 0/7 to 29 6/7 weeks’ gestation. Maternal interviews, clinical measurements, and biospecimens were collected at each study visit. Abstraction of prenatal care and delivery medical records was conducted by a certified abstractor. In the present study, we sequentially excluded participants with missing data on GD, with pregestational diabetes, and with missing county-level radon data due to missing residential information.

### Exposure Data

**Figure 1.**
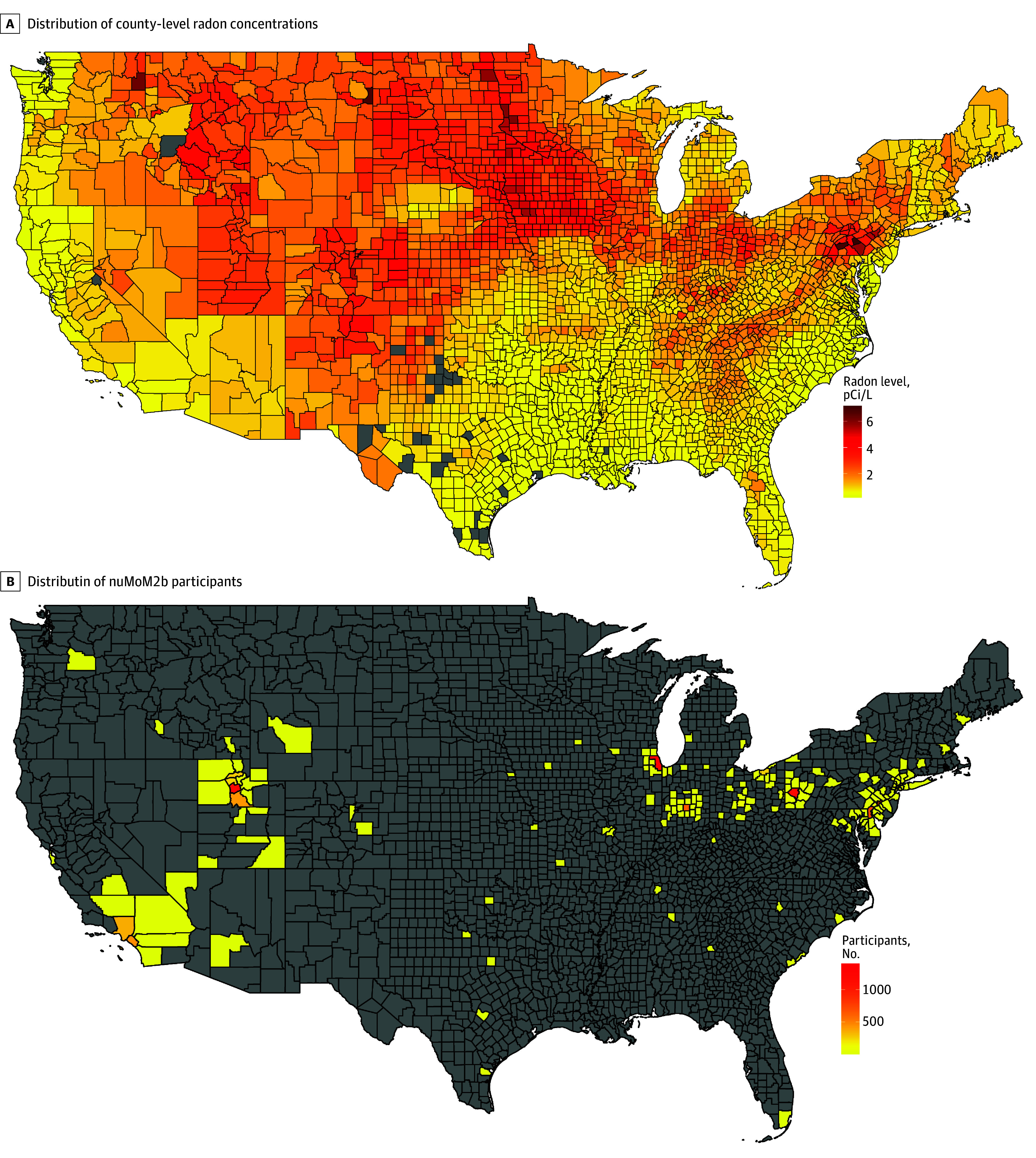
Radon Levels and Study Participants in the Contiguous US, by County Radon-level data are from the Lawrence Berkeley National Laboratory. Counties shaded gray had no radon data available (A) or included no Nulliparous Pregnancy Outcomes Study: Monitoring Mothers-to-Be (nuMoM2b) participants (B). Both maps were created using R, version 4.4.1 (R Project for Statistical Computing). To convert picocuries (pCi) per liter to becquerels (Bq) per cubic meter, multiply by 37.

The estimates of county-level radon for the present study are from the Lawrence Berkeley National Laboratory (LBNL) and have been widely used in previous cohort studies to identify health risks associated with radon.^22,23,24,25^ In brief, researchers from the LBNL and Columbia University used bayesian hierarchical modeling to analyze Environmental Protection Agency (EPA) short- and long-term home radon concentrations^26^ while considering local geological, soil, and meteorological characteristics to provide estimates of the yearly mean indoor radon concentrations for 3079 US counties (Figure 1A).^27^ This method estimates annual mean, living-area radon concentrations from imprecise short-term (days to a few months) radon measurements while considering the spatial variability. Although the county-level radon concentrations were recorded from the late 1980s to the 1990s, the primary predictors of mean radon concentrations in a county are radium or radon content in the ground and soil permeability, both of which generally remain constant over time.^28^ Data were retrieved from a GitHub repository.^29^

Participants’ home addresses were requested at each nuMoM2b visit. Their residential addresses were geocoded, and we linked radon data to individuals by using the Federal Information Processing System codes at each study visit (Figure 1B). For participants missing an address at a study visit, we used the last available address. An estimate of radon exposure was assigned at 4 time points: visits 1, 2, and 3 and the delivery visit.

### Outcome Assessment

The outcome of interest in this study was GD. In the nuMoM2b cohort, the clinical diagnosis of GD was determined based on the glucose tolerance test (GTT) conducted during routine clinical care by 1 of the following criteria^30^: (1) fasting 3-hour 100-g GTT with at least 2 abnormal values (fasting, ≥95 mg/dL; 1 hour, ≥180 mg/dL; 2 hours, ≥155 mg/dL; or 3 hours, ≥140 mg/dL), (2) fasting 2-hour 75-g GTT with at least 1 abnormal value (fasting, ≥92 mg/dL; 1 hour, ≥180 mg/dL; or 2 hours, ≥153 mg/dL), or (3) nonfasting 50-g GTT of 200 mg/dL or greater if no fasting 3-hour or 2-hour GTT was performed (to convert mg/dL to mmol/L, multiply by 0.0555). If no GTT information was available, information on diagnosis of GD from medical record abstraction was used.

### Covariates

Covariates were considered based on the literature. We retrieved demographic information of the mother, including age, self-reported race and ethnicity (Asian, non-Hispanic Black [hereafter, Black], Hispanic, non-Hispanic White [hereafter, White], and other [individuals who did not identify as any of the other categories]), educational attainment, federal poverty level, marital status, and insurance; behavioral and lifestyle variables (body mass index [BMI, calculated as weight in kilograms divided by height in meters squared] at the first study visit, cigarette smoking [yes or no]); season of conception; and newborn sex from the maternal interviews or medical record abstraction. Race and ethnicity were included because studies have indicated racial and ethnic disparities in GD risk.^31,32^

County-level PM_2.5_ data were from the EPA AirData collected at outdoor monitors across the US. The EPA website provides daily summary data by monitor for air pollutants at the county level.^33^ On a given day, the closest air pollutant monitor with data available was used as the data source. For all nuMoM2b participants, the daily mean of PM_2.5_ on each day during pregnancy was downloaded from the EPA website, and a mean value over the course of pregnancy was calculated. PM_2.5_ data from the closest available monitor were linked to participants.

### Statistical Analysis

To best represent the cumulative exposure, we averaged the radon levels from all 4 visits for each participant. We initially planned to analyze the whole sample by an increase of 1 picocurie (pCi)/L (to convert to becquerels per cubic meter, multiply by 37) in radon exposure. However, as the percentage of participants who resided in an area with a radon level above 3 pCi/L was small (3.7%), we characterized radon exposure as a 3-category variable (<1 pCi/L, 1 to <2 pCi/L, and ≥2 pCi/L). Of note, while a level greater than 4 pCi/L is required for mitigation, the EPA recommends that US residents consider mitigation in the home if the radon level is greater than 2 pCi/L.^34^

To describe characteristics of the participants, we calculated means with SDs (or medians with IQRs) for continuous variables and frequencies for categorical variables. Also, we used the analysis of variance test for continuous variables, the Kruskal-Wallis test for nonnormally distributed variables, and the χ^2^ test for categorical variables to assess differences across exposure groups. Multivariable-adjusted logistic regression models were used to estimate odds ratios (ORs) and 95% CIs for the association between county-level radon exposure and GD.

Joint analyses were conducted to assess the potential interactions of radon exposure (<2 vs ≥2 pCi/L) and smoking (ever vs never) associated with risk of GD, adjusting for covariates in model 3. Participants were classified by the joint categories of radon and smoking. We assessed whether the joint effect estimates were larger or smaller than the sum of the individual effect estimates on both the multiplicative and additive scales.^35^ To assess the multiplicative interaction, we incorporated a product term for radon and smoking in the final model. We then directly assessed the coefficient of this product term using the Wald test. To assess the additive interaction, we calculated the relative excess risk due to interaction (RERI) using multiple logistic regression models: ln(*odds*) = β̂_0_ + β̂_1_ × *radon* + β̂_2_ × *smoking* + β̂_3_ × *radon* × *smoking.* Thus, we obtained the RERI_OR_^35^: RERI_OR_ = exp(β̂_1_ + β̂_2_ + β̂_3_) − exp(β̂_1_) − exp(β̂_2_) + 1.

A positive RERI indicated a positive interaction between the 2 exposures, implying synergistic interaction. The SE was estimated using the delta method.^35^ With the same approach, a separate joint analysis was conducted to assess radon and PM_2.5_ (below or above the median) levels in association with GD.

All analyses were conducted from September 2023 to January 2024 using SAS, version 9.4 (SAS Institute Inc). A 2-sided *P* value ≤.10 for tests of multiplicative interaction and a 2-sided *P* value ≤.05 for all other tests were considered statistically significant.

## Results

### Characteristics of the Study Participants

**Figure 2.**
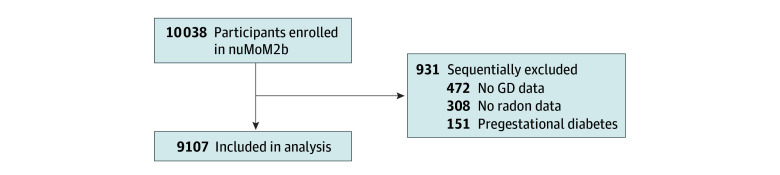
Study Population Flowchart GD indicates gestational diabetes; nuMoM2b, Nulliparous Pregnancy Outcomes Study: Monitoring Mothers-to-Be.

Of the 10 038 individuals included in nuMoM2b, we excluded 472 with missing data on GD, 151 who had pregestational diabetes, and 308 who had missing county-level radon data. As a result, 9107 participants from nuMoM2b were included in this analysis (Figure 2). Of the 9107 participants (median age at first study visit, 27.0 years [IQR, 22.0-31.0 years]; mean [SD] age, 27.0 [5.6] years), 382 (4.2%) developed GD. A total of 362 individuals (4.0%) were Asian; 1207 (13.3%), Black; 1512 (16.6%), Hispanic; 5561 (61.1%), White; and 464 (5.1%), other race and ethnicity. Of 9101 with available data on tobacco use, 3782 (41.6%) had ever used tobacco. In the group exposed to the highest radon concentrations (≥2 pCi/L), participants were more likely to be White and have an educational level of less than high school completion and were less likely to have smoked than groups exposed to less than 2 pCi/L (Table 1).

**Table 1.** Characteristics of the Study Population by County-Level Radon Levels

Characteristic	Participants^a^				*P* value
	Total (N = 9107)	County-level radon concentration, pCi/L^b^			
		<1 (n = 3960)	1 to <2 (n = 2206)	≥2 (n = 2941)	
Radon concentration, mean (SD), pCi/L	1.6 (0.9)	0.8 (0.2)	1.5 (0.2)	2.9 (0.3)	NA
Unique counties, No.	176	48	61	67	NA
Age at study visit 1, median (IQR), y	27.0 (22.0-31.0)	29.0 (24.0-32.0)	27.0 (22.0-31.0)	25.0 (22.0-28.0)	<.001
Age group, y					
13-17	214 (2.4)	85 (2.2)	36 (1.6)	93 (3.2)	<.001
18-35	8290 (91.0)	3523 (89.0)	2016 (91.4)	2751 (93.5)	
>35	602 (6.6)	351 (8.9)	154 (7.0)	97 (3.3)	
Race and ethnicity					
Asian	362 (4.0)	258 (6.5)	57 (2.6)	47 (1.6)	<.001
Hispanic	1512 (16.6)	619 (15.6)	579 (26.3)	314 (10.7)	
Non-Hispanic Black	1207 (13.3)	580 (14.7)	364 (16.5)	263 (8.9)	
Non-Hispanic White	5561 (61.1)	2292 (57.9)	1105 (50.1)	2164 (73.6)	
Other^c^	464 (5.1)	210 (5.3)	101 (4.6)	153 (5.2)	
Married	5521 (60.7)	2473 (62.5)	1074 (48.9)	1974 (67.1)	<.001
Private insurance	6631 (73.3)	3000 (76.1)	1407 (64.6)	2224 (76.0)	<.001
Educational level					
<High school	685 (7.5)	254 (6.4)	155 (7.1)	276 (9.4)	<.001
High school graduate or GED	1040 (11.4)	342 (8.6)	324 (14.7)	374 (12.7)	
Some college	1754 (19.3)	624 (15.8)	430 (19.6)	700 (23.8)	
Associate or technical degree	924 (10.2)	290 (7.3)	209 (9.5)	425 (14.5)	
College graduate	2555 (28.1)	1209 (30.6)	540 (24.6)	806 (27.4)	
>Undergraduate degree	2141 (23.5)	1239 (31.3)	542 (24.6)	360 (12.2)	
FPL, %^d^					
>200	5245 (70.5)	2691 (83.1)	1124 (71.2)	1430 (54.5)	<.001
100-200	1052 (14.1)	249 (7.7)	219 (13.9)	584 (22.3)	
<100	1143 (15.4)	298 (9.2)	235 (14.9)	610 (23.3)	
Ever used tobacco	3782 (41.6)	1949 (49.2)	896 (40.7)	937 (31.9)	<.001
BMI, median (IQR)	24.6 (21.9-28.9)	24.2 (21.8-28.2)	25.1 (22.2-29.9)	24.6 (21.9-29.3)	<.001
BMI group^e^					
<18.5	206 (2.3)	92 (2.4)	48 (2.3)	66 (2.3)	<.001
18.5 to <25	4596 (51.4)	2127 (54.6)	994 (46.8)	1475 (50.6)	
25 to <30	2215 (24.8)	935 (24.0)	562 (26.5)	718 (24.7)	
30 to <35	1063 (11.9)	413 (10.6)	283 (13.3)	367 (12.6)	
≥35	856 (9.6)	331 (8.5)	238 (11.2)	287 (9.9)	
Family history of type 2 diabetes	1876 (21.6)	829 (22.0)	458 (22.4)	589 (20.5)	.20
Season of conception					
Spring	2285 (25.1)	951 (24.0)	588 (26.7)	746 (25.4)	<.001
Summer	2194 (24.1)	889 (22.5)	532 (24.2)	773 (26.3)	
Fall	2247 (24.7)	1051 (26.6)	519 (23.6)	677 (23.0)	
Winter	2376 (26.1)	1068 (27.0)	564 (25.6)	744 (25.3)	
Newborn sex					
Female	4353 (48.6)	1883 (48.3)	1055 (48.4)	1415 (49.0)	.87
Male	4613 (51.5)	2013 (51.7)	1124 (51.6)	1476 (51.1)	
Systolic blood pressure, mean (SD), mm Hg	109.0 (10.8)	109.2 (10.6)	109.2 (10.4)	108.6 (11.4)	.04
Daily mean PM_2.5_ values during pregnancy, mean (SD), μg/m^3^	10.5 (1.8)	10.8 (1.3)	10.5 (1.5)	10.1 (2.3)	<.001

Abbreviations: BMI, body mass index (calculated as weight in kilograms divided by height in meters squared); FPL, federal poverty level; GED, General Educational Development; NA, not applicable; pCi, picocurie; PM_2.5_, fine particulate matter air pollutants.

^a^ Data are presented as number (percentage) of participants unless otherwise indicated. Proportions may not sum to 100% due to rounding and because certain variables had small numbers of participants with missing data.

^b^ To convert pCi per liter to becquerels per cubic meter, multiply by 37.

^c^ Represents individuals who did not identify as any of the other categories.

^d^ Based on 2013 federal poverty guidelines.

^e^ A BMI less than 18.5 is considered underweight; 18.5 to less than 25, healthy weight; 25 to less than 30, overweight; 30 to less than 35, obesity; and 35 or higher, morbid obesity.

### Radon Exposure and GD

The mean (SD) county-level radon concentration was 1.6 (0.9) pCi/L. After adjusting for potential confounders (Table 2), participants from the highest radon group (≥2 pCi/L) had higher odds of developing GD compared with those from the lowest radon group (<1 pCi/L) (OR, 1.37; 95% CI, 1.02-1.84). After additional adjustment for PM_2.5_, results were not appreciably different (OR, 1.36; 95% CI, 1.00-1.86).

**Table 2.** Association Between Radon Exposure and Gestational Diabetes^a^

Model	Odds ratio (95% CI)^b^		
	<1 pCi/L	1 to <2 pCi/L	≥2 pCi/L
1^c^	1 [Reference]	1.03 (0.79-1.34)	1.20 (0.94-1.54)
2^d^	1 [Reference]	1.00 (0.73-1.37)	1.37 (1.02-1.84)
3^e^	1 [Reference]	0.97 (0.70-1.35)	1.36 (1.00-1.86)

Abbreviation: pCi, picocurie.

^a^ N =  9107. There were 169 cases among 3960 participants in the group with radon exposure less than 1 pCi/L (4.3%); 91 among 2206 in the group with radon exposure of 1 to less than 2 pCi/L (4.1%); and 122 among 2941 in the group with radon exposure of 2 pCi/L or higher (4.1%).

^b^ To convert pCi per liter to becquerels per cubic meter, multiply by 37.

^c^ Adjusted for age and race and ethnicity.

^d^ Additionally adjusted for the following factors in Table 1: educational level, marital status, insurance, percentage of federal poverty level, tobacco smoking, body mass index at visit 1, systolic blood pressure, season of conception, and infant sex.

^e^ Additionally adjusted for fine particulate matter air pollutants.

In analyses restricted to individuals with specific lifestyle factors, there was no association between highest vs lowest radon exposure and GD among never smokers (OR, 1.31; 95% CI, 0.84-2.05) and individuals with healthy weight (BMI ≤25) (OR, 1.38; 95% CI, 0.80-2.39). The proportions of participants who had missing county-level radon data (308 [3.1%]) or missing GD data (472 [4.7%]) and were excluded from the primary analysis were small. In our sensitivity analyses, we used multiple imputation for GD. Additionally, we assigned individuals with missing radon data to 2 extreme scenarios: all to the group exposed to 2 or more pCi/L or all to the group exposed to less than 1 pCi/L. There was no association when multiple imputation was used (the OR changed from 1.36 [95% CI, 1.00-1.86] to 1.27 [95% CI, 0.91-1.63]). For the missing radon sensitivity analyses in either scenario, the association of interest was essentially unchanged from the main analysis.

### Joint Associations

**Figure 3.**
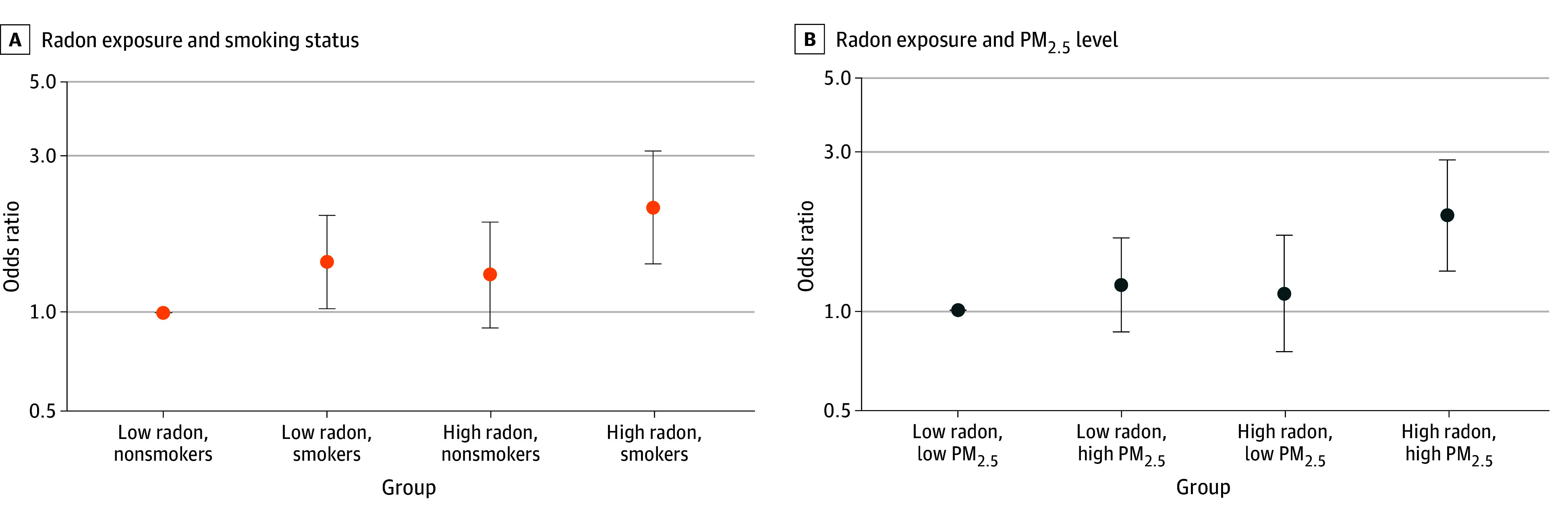
Multivariable Odds Ratios of Developing Gestational Diabetes by Smoking Status, Radon Exposure, and Fine Particulate Matter Air Pollutants (PM_2.5_) Exposure A, Nonsmokers with low radon exposure was the reference group. B, The group with low radon and low PM_2.5_ exposure was the reference group. High radon level was considered to be 2 picocuries (pCi)/L or greater and low, less than 2 pCi/L (to convert to becquerels per cubic meter, multiply by 37). High PM_2.5_ level was considered to be above the median and low, below the median.

In the joint analysis (Figure 3A) adjusting for the same covariates as in model 3, the odds of developing GD were more pronounced in ever smokers residing in high-radon counties (OR, 2.09; 95% CI, 1.41-3.11) compared with never smokers in low-radon counties. The OR for developing GD in the low-radon level and ever smoker group was 1.43 (95% CI, 1.03-1.98) and in the high-radon level and never smoker group was 1.31 (95% CI, 0.90-1.89). However, we did not detect a statistically significant interaction on the multiplicative or additive scales.

Additionally, we classified PM_2.5_ by its median level (10.34 μg/m^3^ [IQR, 9.34-11.51 μg/m^3^]) and assessed the joint associations of radon and PM_2.5_ with GD (Figure 3B). The odds of GD were elevated in participants exposed to high radon and high PM_2.5_ levels (OR, 1.93; 95% CI, 1.31-2.83) compared with those exposed to low radon and low PM_2.5_ levels. The ORs were not statistically significant in the group with low radon and high PM_2.5_ levels (OR, 1.19; 95% CI, 0.86-1.65) or the groups with high radon and low PM_2.5_ levels (OR, 1.12; 95% CI, 0.75-1.68). We did not detect a statistically significant interaction on the multiplicative or additive scales.

## Discussion

In this large cohort study of nulliparous pregnant individuals, we found a significant association between county-level radon exposure and the odds of GD, particularly in subgroups with higher exposure to radon and either high PM_2.5_ level or smoking. Our findings are consistent with results from a previous study assessing the association between ambient particle radioactivity measured by PM_2.5_ gross β and the incidence of GD among pregnant women in Massachusetts.^15^ The observed association is biologically plausible. Radon and RDPs emit alpha particles that can induce oxidative stress and promote inflammation,^36^ processes associated with chronic inflammatory diseases,^37,38,39^ such as hypertensive disorders of pregnancy. Additionally, radon exposure may lead to DNA damage and epigenetic changes in placental cells^40^ as well as mitochondrial dysfunction.^41^ Together, these combined pathways may contribute to placental vascular dysfunction by disrupting blood flow and nutrient exchange, creating hypoxic conditions that promote insulin resistance.^42,43^ Consequently, these disruptions can impair glucose metabolism in the mother, increasing the risk of developing GD. To our knowledge, this is the first study to examine the association between radon exposure and the risk of GD. While the Massachusetts study^15^ focused on the radionuclide components of ambient particles (with radon as a primary source of radiation), our study explored overall radon exposure, encompassing both its gaseous and attached forms.

We observed more pronounced odds for GD among participants jointly exposed to smoking and radon; however, the interactions were not statistically significant. Smokers may inherently possess a higher baseline risk for GD due to mechanisms such as insulin resistance and inflammation.^5^ However, smoking and radon exposure may influence distinct inflammatory pathways; smoking primarily induces neutrophilic inflammation, while radon exposure affects eosinophilic cells.^44^ This might partially explain the lack of an observed joint association between these exposures in the current study. We cannot rule out an interaction between radon exposure and smoking with GD, although it was not statistically significant in this cohort. Further investigation is needed.

We also observed an increasing pattern in the odds of GD among participants exposed to higher levels of both radon and PM_2.5_ during pregnancy. Similarly, although not statistically significant in this study, the interaction of these 2 air pollutants is biologically plausible. In addition to promoting systemic oxidative stress^17^ and inflammation,^16^ research has shown that radon and radioactive PM can cause significant cellular and biochemical damage.^10,45^ A previous meta-analysis of 10 cohort studies found an association between PM_2.5_ exposure and an elevated risk of type 2 diabetes.^46^ Notably, some epidemiologic evidence has demonstrated a synergistic interaction of radon and air pollution with various health outcomes, including childhood leukemia,^47^ cardiovascular disease mortality,^48^ and respiratory and all-cause mortality.^45^

### Future Implications

This study provides insight for generating hypotheses regarding radon and the risk of GD. To test the hypotheses, it is vital to conduct studies that incorporate individual-level indoor radon exposure data. This approach will not only reduce measurement errors but also facilitate the exploration of the underlying mechanisms. For example, individual-level radon data provide the data granularity necessary to examine the associations between radon and metabolic biomarkers, which can affect the onset of GD. Additionally, more advanced methods for indoor radon assessment should be considered; for example, the glass-based retrospective radon exposure reconstruction detector presents a novel and practical tool for assessing accumulated indoor radon exposure.^49^ Since young adults often move frequently, using a glass object, such as a photo frame or an accessory, may provide more accurate cumulative radon exposure compared with radon detectors placed in a current house. Furthermore, our joint analyses of radon and either smoking or PM_2.5_ provide important preliminary data for future studies. For instance, studies aiming to investigate the health effects of radon exposure among never smokers can potentially yield robust findings by minimizing confounding from smoking. This strategy is supported by studies related to the impact of radon exposure on various diseases^50,51,52^ that indicated that never smokers, who are at a lower baseline risk for these health conditions, experienced a more substantial relative increase in risk from radon exposure compared with smokers.

### Strengths and Limitations

Our study has several strengths. First, we included a large and diverse sample of approximately 10 000 pregnant individuals. The diversity enhanced the generalizability of our findings. Second, our assessment of GD outcomes was based on robust clinical diagnoses. Third, the study benefited from a comprehensive set of individual-level variables, including insurance status and BMI. The availability of these variables helped mitigate confounding bias. Fourth, this cohort allowed us to examine the interaction between radon and either smoking or PM_2.5_, furthering the understanding of their possible combined impact on GD risk.

There are also several limitations worth noting. First, as demonstrated in previous reviews of studies on radon exposure and adverse health outcomes conducted by some of us,^53,54,55^ ecological measures of radon exposure may fail to account for temporal and spatial variations within a county. The average radon level in a county might not reflect an individual’s radon exposure. Therefore, individual-level residential measurements of radon exposure (eg, household mitigation practices, presence of a basement) are crucial for enhancing the precision of exposure assessment. Second, the county-level radon measurements used in this study were taken during the 1990s, which may not represent the radon level during the study. While county-level radon levels tend to remain stable over time,^56^ changes in some housing parameters, such as air conditioner use and radon mitigation practices, may have somewhat influenced the pattern of county-level radon concentrations and introduced measurement errors. However, we compared the LBNL data with more recent sources, such as data from the Environmental Public Health Tracking Network,^57^ and found good agreement between these 2 radon sources. Third, we acknowledge that a small portion of participants had missing data for either radon exposure or GD. However, as shown in our sensitivity analysis, this did not alter our conclusions. Fourth, residual and/or unknown confounding cannot be ruled out. In particular, information on residential type (eg, single-family house vs apartment or condominium) and years of residing in the current home was not available, which further highlights the importance of individual-level exposure assessment.

## Conclusions

Findings from this cohort study based on county-level radon exposure support the hypothesis that radon may be associated with the risk of GD in nulliparous pregnant individuals. This study provides a foundation for future research focusing on individual-level indoor radon measurement to confirm the findings and explore the underlying biological mechanisms.

## Notes

Supplement.
Data Sharing Statement
