# Supplementary material for: Radon Exposure and Gestational Diabetes
Source: JAMA Netw Open. 2025 Jan 10;8(1):e2454319. doi: 10.1001/jamanetworkopen.2024.54319 (PMC11724344; doi:10.1001/jamanetworkopen.2024.54319)
Supplement: Supplement. — Data Sharing Statement [file jamanetwopen-e2454319-s001.pdf]

Data Sharing Statement

Zhang. Radon Exposure and Gestational Diabetes. *JAMA Netw Open*. Published January 10, 2025. doi:10.1001/jamanetworkopen.2024.54319

Data
Data available: Yes
Data types: Deidentified participant data
How to access data: Deidentified patient data is available on DASH https://dash.nichd.nih.gov/study/226675
When available: With publication

Supporting Documents
Document types: None

Additional Information
Who can access the data: Please follow the instructions on the DASH website to access data. https://dash.nichd.nih.gov/study/226675
Types of analyses: Please follow the instructions on the DASH website to access data. https://dash.nichd.nih.gov/study/226675
Mechanisms of data availability: Please follow the instructions on the DASH website to access data. https://dash.nichd.nih.gov/study/226675
